# Supplementary material for: Strong Hydrogen Bonds in Acetylenedicarboxylic Acid Dihydrate
Source: Int J Mol Sci. 2022 May 31;23(11):6164. doi: 10.3390/ijms23116164 (PMC9181247; doi:10.3390/ijms23116164)
Supplement: Supplementary file 1 [file ijms-23-06164-s001.zip › ijms-1722134-supplementary.pdf]

Supplementary

Urban Novak, Amalija Golobič, Natalija Klančnik, Vlasta Mohaček-Grošev, Jernej Stare and Jože Grdadolnik

Table S1. Crystal data, data collection and refinement of α, β1, β2 at 293, αD, β1D and β2D.

Crystal structure	**α**	**β1**	**β2**	**αD**	**β1D**	**β2D**
Formula of compound	$C_4H_2O_4 \cdot 2H_2O$	$C_4H_2O_4 \cdot 2H_2O$	$C_4H_2O_4 \cdot 2H_2O$	$C_4D_2O_4 \cdot 2D_2O$	$C_4D_2O_4 \cdot 2D_2O$	$C_4D_2O_4 \cdot 2D_2O$
M_r	150.08	150.08	150.08	156.12	156.12	156.12
Crystal system	monoclinic	monoclinic	monoclinic	monoclinic	monoclinic	monoclinic
Space group	$P2_1/c$, No. 14	$P2_1/c$, No. 14	$P2_1/c$, No. 14	$P2_1/c$, No. 14	$P2_1/c$, No. 14	$P2_1/c$, No. 14
a [Å]	7.9831(7)	7.0611(6)	6.9954(4)	7.9965(6)	7.0918(6)	7.0143(5)
b [Å]	3.8666(4)	5.4510(4)	5.4828(4)	3.8710(3)	5.4471(5)	5.4727(4)
c [Å]	11.0665(12)	9.8228(13)	9.7714(9)	11.0626(8)	9.8523(14)	9.7669(12)
β [°]	97.909(9)	115.867(8)	117.155(6)	98.042(7)	115.886(9)	117.175(8)
V [Å^3]	338.35(6)	340.20(6)	333.47(4)	339.07(4)	342.40(6)	333.54(5)
No. of formula units, Z	2	2	2	2	2	2
D_X [Mg m^{-3}]	1.473	1.465	1.495	1.529	1.514	1.555
F_{000}	156	156	156	156	156	156
X-Ray radiation	Cu K_α	Cu K_α	Mo K_α	Mo K_α	Mo K_α	Mo K_α
Wavelength, λ [Å]	1.54184	1.54184	0.71073	0.71073	0.71073	0.71073
μ [mm^{-1}]	1.293	1.286	0.147	0.144	0.143	0.147
T [K]	293(2)	263(2)	150(2)	293(2)	263(2)	150(2)
Colour	colourless	colourless	colourless	colourless	colourless	colourless
Dimensions of crystal [mm]	0.32x0.29x0.09	0.35x0.30x0.25	0.31x0.27x0.08	0.34x0.30x0.15	0.42x0.30x0.25	0.42x0.30x0.25
No. of measured reflections	1314	1367	1775	1805	3065	3045

No. of unique reflections	688	690	880	894	925	905
R_{int}, [%]	0.0173	0.0116	0.0241	0.0158	0.0349	0.0624
No of (F^2 > 2.0σ(F^2)) reflections	571	635	677	621	591	609
Θ maximal [°]	75.02	74.97	30.16	30.37	30.23	30.11
R[F^2 > 2σ (F^2)]	0.0550	0.0366	0.041	0.043	0.0486	0.0451
$wR(F^2)$	0.1519	0.1044	0.1104	0.1045	0.1163	0.1155
Goof, *S*	1.088	1.058	1.092	1.042	1.087	1.100
No. of contributing reflections	688	690	880	894	925	905
No. of parameters	58	58	58	58	58	58
(Δ/*σ*) maximal	0.002	0.000	0.000	0.000	0.000	0.000
(Δ*ρ*)– maximal [e $Å^{-3}$]	0.198	0.218	0.252	0.206	0.185	0.232
(Δ*ρ*)– minimal $Å^{-3}$]	–0.225	–0.150	–0.230	–0.143	–0.224	–0.253

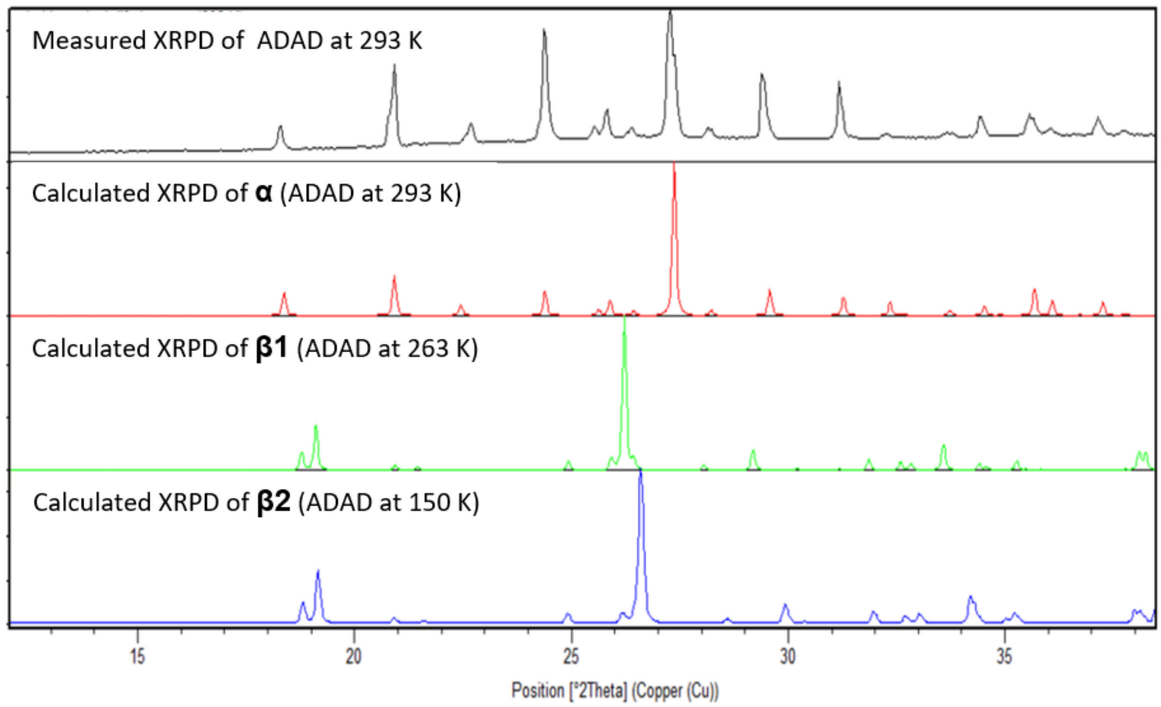

Figure S1. X-ray powder diffraction patterns (XRPD) of ADAD.

Table S2. Bond lengths and angles.

Bond length (Å)	**α**	**β1**	**β2**	**αD**	**β1D**	**β2D**
C1—C1^{i}	1.181(4)	1.185(2)	1.189(3)	1.183(3)	1.189(3)	1.191(3)
C1—C2	1.465(2)	1.4615(16)	1.465(2)	1.464(2)	1.461(2)	1.465(2)
C2—O2	1.204(2)	1.2091(16)	1.2143(18)	1.2046(19)	1.209(2)	1.2104(19)
C2—O1	1.285(3)	1.2892(17)	1.2991(19)	1.287(2)	1.294(2)	1.295(2)
O1—H1	0.95(4)	0.92(2)	0.91(3)	0.93(3)	0.94(3)	0.89(2)
O3—H2	0.77(5)	0.93(3)	0.81(3)	0.80(3)	0.81(3)	0.79(2)
O3—H3	0.99(3)	0.88(2)	0.97(2)	0.88(3)	0.89(3)	0.99(2)
Bonding angle (°)	**α**	**β1**	**β2**	**αD**	**β1D**	**β2D**
C1^{i}—C1—C2	176.2(3)	176.85(19)	176.7(2)	176.1(2)	176.7(3)	176.6(2)
O2—C2—O1	125.26(16)	125.66(11)	125.78(13)	125.45(14)	125.47(15)	125.73(15)
O2—C2—C1	120.42(17)	121.05(11)	120.87(13)	120.55(15)	121.32(16)	121.03(14)
O1—C2—C1	114.32(16)	113.29(11)	113.35(13)	114.00(15)	113.20(16)	113.24(15)
C2—O1—H1	110(2)	107.2(15)	112.2(17)	108.8(15)	110.9(15)	110.6(15)
H2—O3—H3	105(3)	109(2)	107(2)	112(3)	114(2)	107.1(18)

(i) −x, −y, −z+2

Table S3. Geometric parameters of hydrogen bonds in protic (upper table) and deuterated (lower table) ADAD .

**α**	X—H (Å)	H···Y (Å)	X···Y (Å)	X—H···Y (°)
O1—H1···O3	0.95(3)	1.60(3)	2.542(2)	172(3)
O3—H2···O2^{i}	0.77(5)	2.27(5)	2.904(2)	141(4)
O3—H2···O3^{i}	0.77(5)	2.59(4)	3.098(3)	125(3)
O3—H3···O2^{ii}	1.00(3)	1.81(3)	2.774(2)	163(3)
**β1**				
O1—H1···O3	0.91(2)	1.63(3)	2.5394(18)	174(3)
O3—H2···O2^{i}	0.93(3)	2.01(3)	2.8671(18)	152(3)

O3—H2···O3^{i}	0.93(3)	2.57(3)	3.1329(16)	120(2)
O3—H3···O2^{ii}	0.88(2)	1.92(2)	2.7724(16)	164(2)
**β2**				
O1—H1···O3	0.92(3)	1.62(3)	2.5328(19)	176(2)
O3—H2···O2^{i}	0.81(3)	2.10(3)	2.8513(18)	154(3)
O3—H2···O3^{i}	0.81(3)	2.614(3)	3.1100(16)	121(2)
O3—H3···O2^{ii}	0.97(2)	1.80(2)	2.7654(16)	172(2)

**αD**	X—D (Å)	D···Y (Å)	X···Y (Å)	X—D···Y (°)
O1—D1···O3	0.93(2)	1.62(2)	2.552(2)	174(2)
O3—D2···O2^{i}	0.80(3)	2.20(3)	2.909(2)	149(2)
O3—D2···O3^{i}	0.80(3)	2.55(3)	3.088(3)	126(3)
O3—D3···O2^{ii}	0.87(3)	1.91(3)	2.782(2)	175(3)
**β1D**				
O1—D1···O3	0.94(3)	1.61(3)	2.553(2)	179(4)
O3—D2···O2^{i}	0.81(3)	2.16(3)	2.882(2)	149(3)
O3—D2···O3^{i}	0.81(3)	2.59(3)	3.137(2)	126(3)
O3—D3···O2^{ii}	0.89(3)	1.91(3)	2.782(2)	167(3)
**β2D**				
O1—D1···O3	0.89(3)	1.65(3)	2.542(2)	178(3)
O3—D2···O2^{i}	0.79(2)	2.13(2)	2.860(2)	153(2)
O3—D2···O3^{i}	0.79(2)	2.617(3)	3.103(2)	121(2)
O3—D3···O2^{ii}	0.99(2)	1.80(2)	2.770(2)	169(2)

(i) 1–x,1/2+y,3/2–z; (ii) x, –1/2–y,–1/2+z;

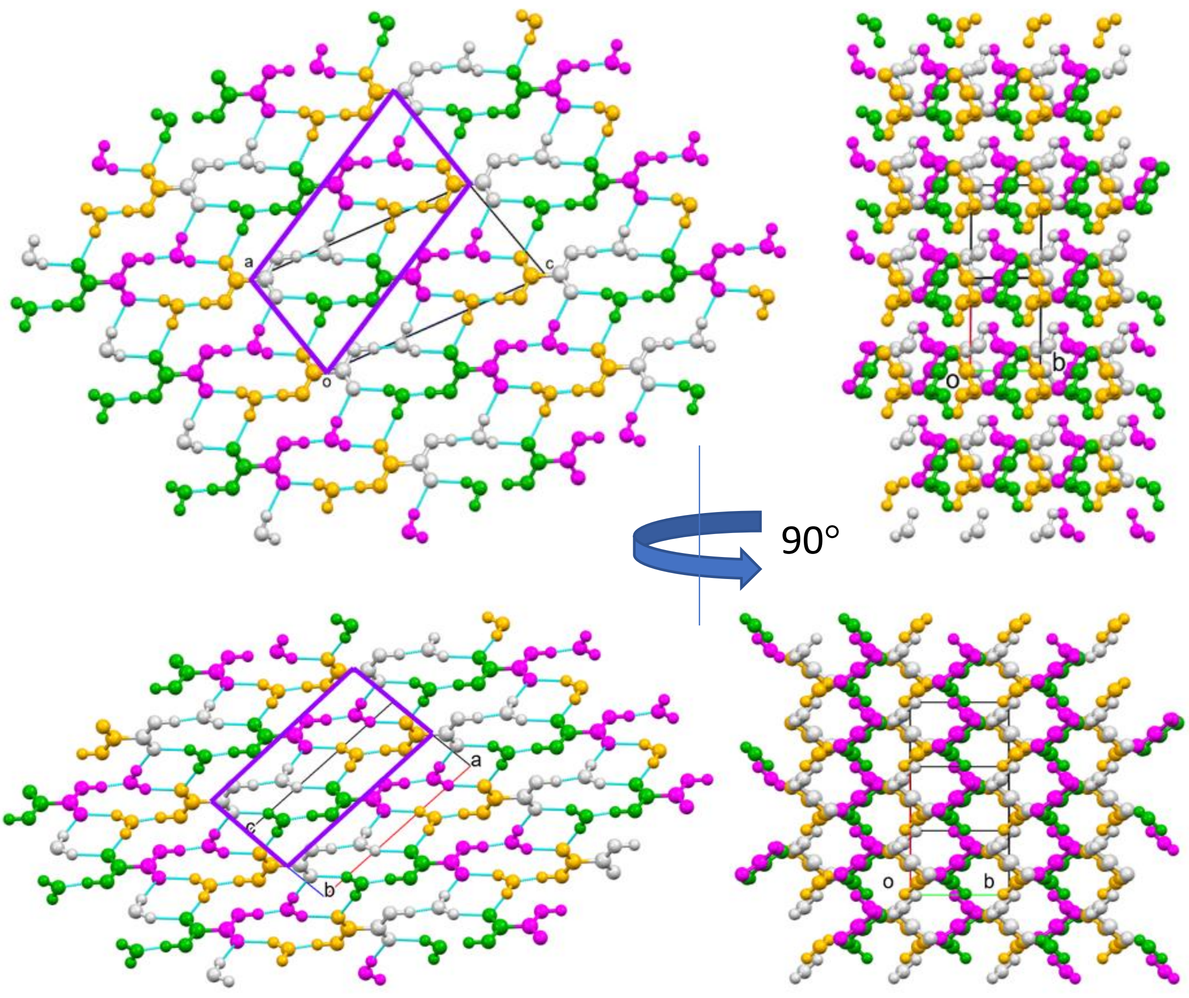

Figure S2. Presentation of crystal packing of molecules in OADH using colouring for symmetry operations: asymmetric unit is drawn in light grey, inversion symmetry in yellow, 2-fold screw axis.

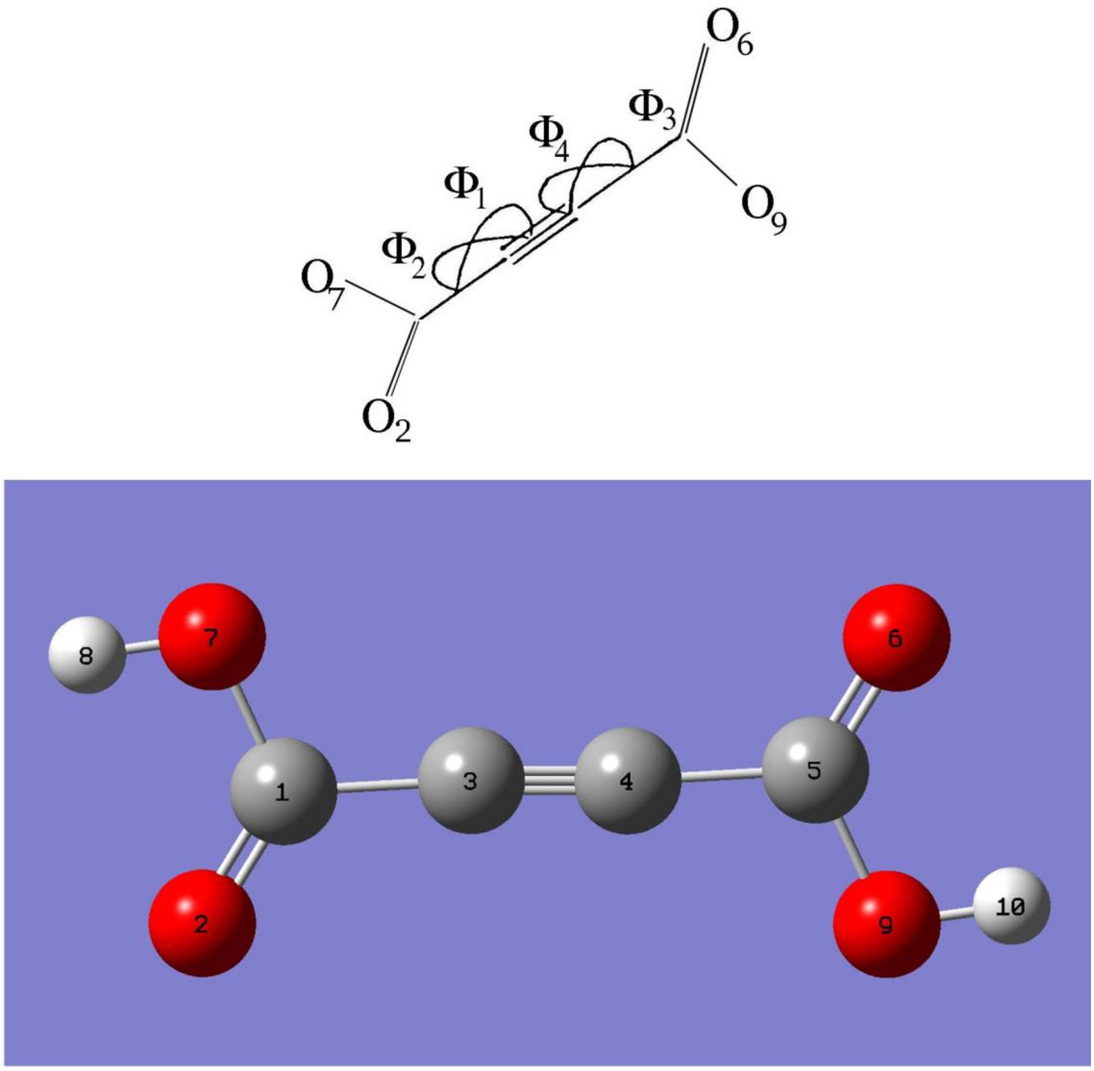

Figure S3. The definition and assignment of torsion angles and atom numbers used in QM calculations.

Table S4. Definition of internal coordinates in *trans* acetylenedicarboxylic acid. Atom numbering is shown in Supplementary Figure S3.

Deformations of bond stretching coordinates

1. $\Delta T = \Delta(C_3 \equiv C_4)$
2. $\Delta D_1 = \Delta(C_1{=}O_2)$
3. $\Delta D_2 = \Delta(C_5{=}O_6)$
4. $\Delta R_1 = \Delta(C_1 - C_3)$
5. $\Delta R_2 = \Delta(C_4 - C_5)$
6. $\Delta S_1 = \Delta(C_1 - O_7)$
7. $\Delta S_2 = \Delta(C_5 - O_9)$
8. $\Delta l_1 = \Delta(O_7 - H_8)$
9. $\Delta l_2 = \Delta(O_9 - H_{10})$
10. $\Delta \varepsilon_1 = \Delta(C_1 - O_7 - H_8)$
11. $\Delta \varepsilon_2 = \Delta(C_5 - O_9 - H_{10})$
12. $\Delta \eta_1 = \Delta(O_2 - C_1 - O_7)$
13. $\Delta \eta_2 = \Delta(O_6 - C_5 - O_9)$
14. $\Delta \kappa_1 = \Delta(O_2 - C_3 - C_1)$
15. $\Delta \kappa_2 = \Delta(O_6 - C_5 - C_4)$
16. $\Delta \lambda_1 = \Delta$(out of plane $C_1 - O_7$ bending)
17. $\Delta \lambda_2 = \Delta$(out of plane $C_5 - O_9$ bending)
18. $\Delta \Phi_1 = \Delta$(linear bending $O_2 - C_1 - C_3 - C_4$ in the molecular plane)
19. $\Delta \Phi_2 = \Delta$(linear bending $O_2 - C_1 - C_3 - C_4$ perpendicular to the molecular plane)
20. $\Delta \Phi_3 = \Delta$(linear bending $O_6 - C_5 - C_4 - C_3$ in the molecular plane)
21. $\Delta \Phi_4 = \Delta$(linear bending $O_6 - C_5 - C_4 - C_3$ perpendicular to the molecular plane)
22. $\Delta \tau OH_1 = \Delta$(torsion $H_8 - O_7 - C_1 - C_3$)
23. $\Delta \tau OH_2 = \Delta$(torsion $H_{10} - O_9 - C_5 - C_4$)
24. $\Delta \tau COOH = \Delta$(torsion $O_6 - C_5 - C_4 - O_7$ + torsion $O_2 - C_1 - C_3 - O_9$)

Table S5. Definition of symmetrized coordinates for *trans* acetylenedicarboxylic acid with **C_{2h}** symmetry.

9 A_g

1. $S_1 = \Delta T$
2. $S_2 = \Delta D_1 + \Delta D_2$
3. $S_3 = \Delta R_1 + \Delta R_2$
4. $S_4 = \Delta S_1 + \Delta S_2$
5. $S_5 = \Delta l_1 + \Delta l_2$
6. $S_6 = \Delta \varepsilon_1 + \Delta \varepsilon_2$
7. $S_7 = \Delta \eta_1 + \Delta \eta_2$
8. $S_8 = \Delta \kappa_1 + \Delta \kappa_2$
9. $S_9 = \Delta \Phi_1 + \Delta \Phi_3$

3 B_g

10. $S_{10} = \Delta \lambda 1 - \Delta \lambda 2$
11. $S_{11} = \Delta \Phi_2 + \Delta \Phi_4$
12. $S_{12} = \Delta \tau OH_1 - \Delta \tau OH_2$

4 A_u

13. $S_{13} = \Delta \lambda 1 + \Delta \lambda 2$
14. $S_{14} = \Delta \tau OH_1 + \Delta \tau OH_2$
15. $S_{15} = \Delta \Phi_2 - \Delta \Phi_4$
16. $S_{16} = \Delta \tau COOH$

8 B_u

17. $S_{17} = \Delta D1 - \Delta D2$
18. $S_{18} = \Delta R_1 - \Delta R_2$
19. $S_{19} = \Delta S_1 - \Delta S_2$
20. $S_{20} = \Delta l_1 - \Delta l_2$
21. $S_{21} = \Delta \varepsilon_1 - \Delta \varepsilon_2$
22. $S_{22} = \Delta \eta_1 - \Delta \eta_2$
23. $S_{23} = \Delta \kappa_1 - \Delta \kappa_2$
24. $S_{24} = \Delta \Phi_1 - \Delta \Phi_3$

Table S6. Unscaled normal modes of *trans* acetylenedicarboxylic acid with $\mathbf{C_{2h}}$ symmetry with potential energy distribution (PED) among symmetrized coordinates calculated by BALGA† program are compared with the observed bands of crystal. Contributions greater than 10% are given. Torsional frequency of carboxyl groups is calculated as imaginary.

symmetry $\mathbf{C_{2h}}$	observed Raman crystal 293 K	observed infrared crystal 295 K	calculated	PED (%)	approximate description
$\mathbf{A_g}$					
	3402			100 S_5	water sym. OH str.
	2246		2365	84 S_1 + 16 S_3	C≡C stretch.
	2004 br		3761	89 S_2	sym. O-H stretching
	1688,1649	inactive	1816	45 S_6 + 28 S_4 + 19 S_7	C=O sym. str.
	1454,1445		1374	46 S_4 + 41 S_6	COH bend.+C-O str.
	1332,1288		1188	33 S_7 + 22 S_3 + 22 S_4	C-O str. + COH bend
	758		734	56 S_8 + 35 S_9	O=C-O scissor
	609		615	49 S_3 + 44 S_7	O=C-C bend.
	415		358	63 S_9 + 36 S_8	C-C str.+O=C-C sciss.
	212		204		in pl. CC≡CC bend.
$\mathbf{B_g}$	3470 sh				water OH str
	808, 794		767	88 S_{10}	out of plane C-O bend
	637		627	93 S_{12}	OH torsion
	315,285	inactive	327	80 S_{11}	out of pl.CC≡CC bend
$\mathbf{A_u}$		3508			water asym. OH str.
		758	767	91 S_{13}	out of plane C-O bend
		1071	614	97 S_{14}	OH torsion
	inactive		120	97 S_{15}	o.of pl. CC≡CC bend
			35i	100 S_{16}	COOH torsion
$\mathbf{B_u}$		3382			water sym. OH str.
			3761	100 S_{20}	antisym.O-H stretch.
		1968 br			
		1647,1602	1823	87 S_{17}	antisym.C=O sym. str
		1435	1401	35 S_{21} + 24 S_{19} + 23 S_{18}	COH bend.+C-O str.
		1257,1271	1196	52 S_{21} + 28 S_{19} + 15 S_{18}	COH bend.+C-O str
	inactive	994	986	55 S_{18} + 38 S_{19}	C-C str.+C-O str
			599	47 S_{22} + 35 S_{23}	O=C-O scissor
			542	46 S_{23} + 40 S_{22}	O=C-C bend
			81	86 S_{24}	in pl. CC≡CC bend.

† G. Keresztury, G. Jalsovszky J. Mol. Struct. 10 (1971) 304.
H. Rostkowski, L. Lapinski, M. Nowak Vib. Spectrosc. 49 (2009) 43.

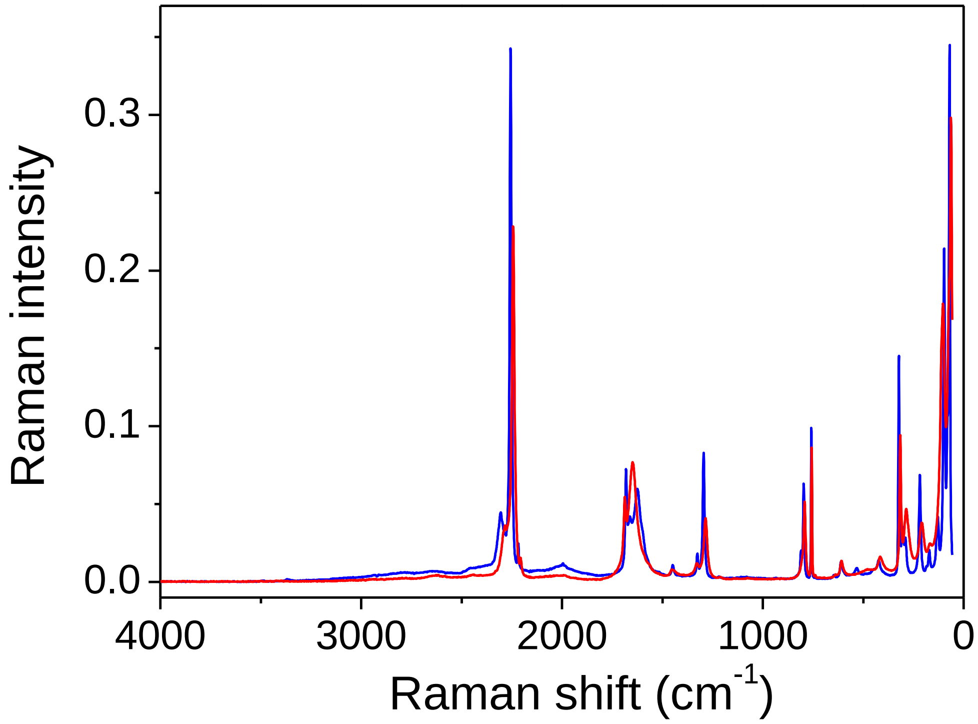

Figure S4. The FT-Raman spectrum of ADAD recorded at T=25°C (red line) and T=-110°C (blue line).

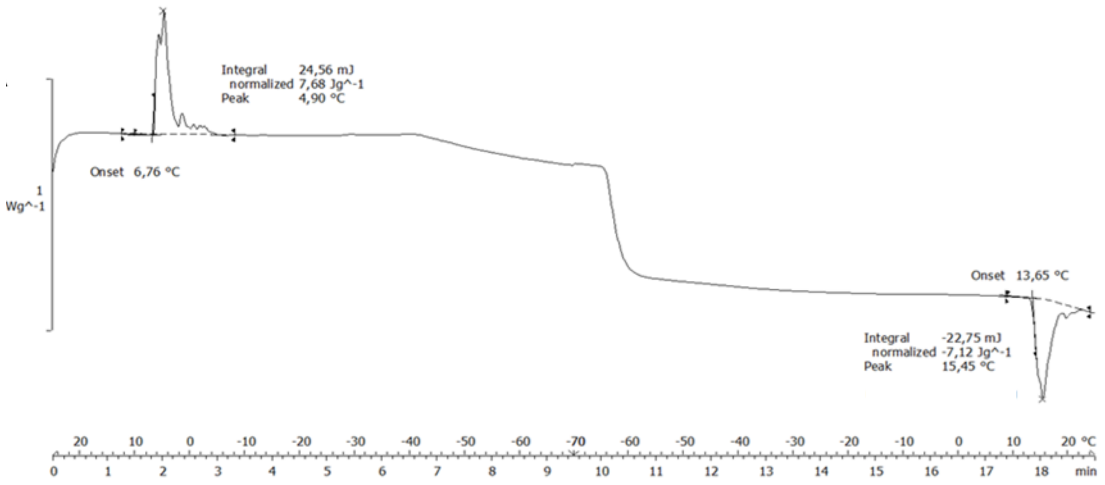

Figure S5. DSC curve of ADAD.

Additional X-ray data

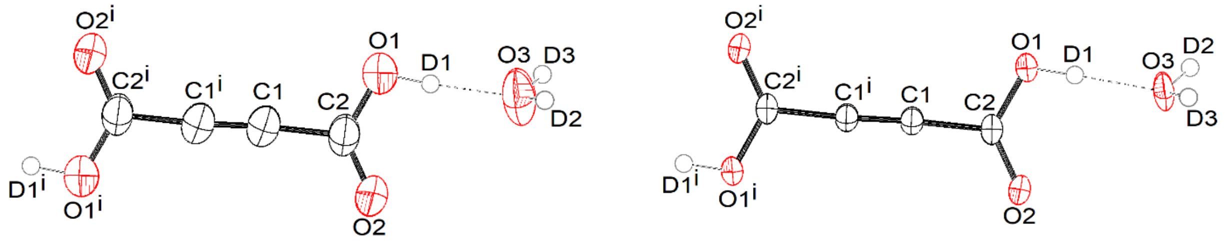

Figure S6. Ortep drawings with atom labelling scheme of water and acetylenecarboxylic acid molecules in αD (left) and β2D (right) structure. Displacement ellipsoids are at 50% probabilty level. (i: –x, –y, –z+2) .

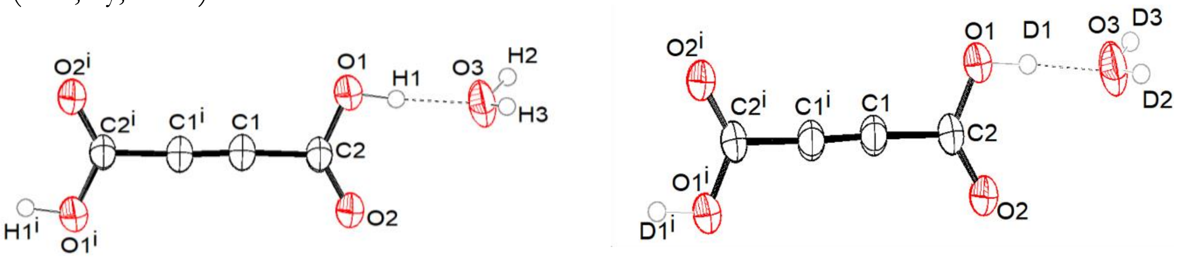

Figure S7. Ortep drawings with atom labelling scheme of water and acetylenecarboxylic acid molecules n β1 (left) and β1D (right) structure. Displacement ellipsoids are at 50% probabilty level. (i: –x, –y, –z+2).

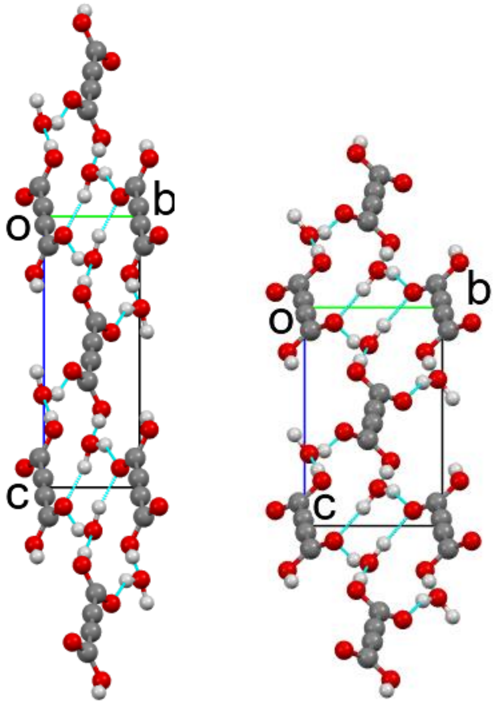

Figure S8. Drawings of molecular packing of α (left) and β2 (right) structure viewed along *a* axis. Blue lines represent hydrogen bonds.

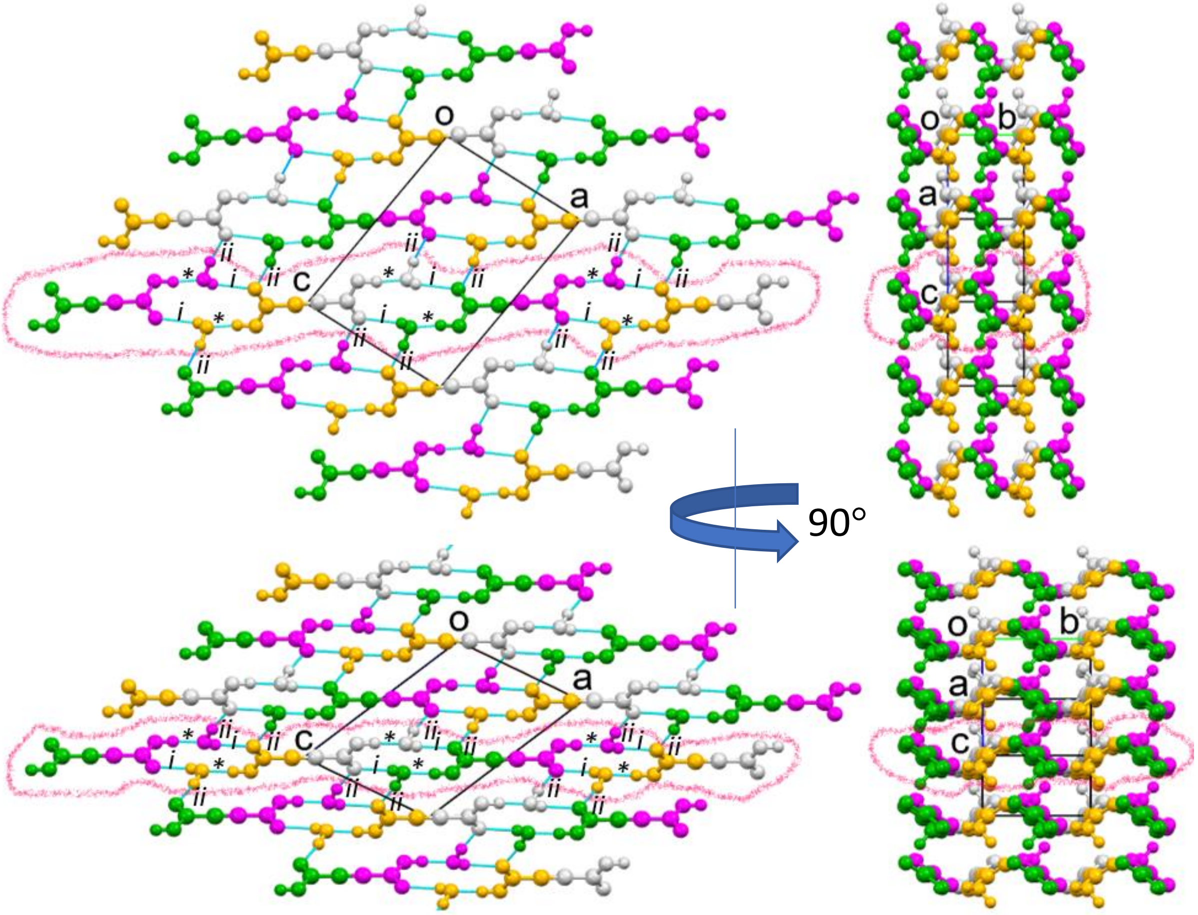

Figure S9. Presentation of crystal packing of molecules in ADAD using colouring for symmetry operations: asymmetric unit is drawn in light grey, inversion symmetry in yellow, 2-fold screw axis in green, and *c* glide plane in pink. Left drawings represent a view on structures of α (top) and β2 (bottom) along *b* axis and right drawings a view on the structures rotated for 90° with *b* axis lying in plane of paper.
in green, and *c* glide plane in pink. Left drawings represent a view on structures of α (top) OXACDH28 and β (bottom) OXACDH29 along *b* axis and right drawings a view on the structures rotated for 90° with *b* axis lying in plane of paper. The purple lines represent unit cells after cell transformation on the same coordinate system.
OXACDH28, OXACDH29: [6]

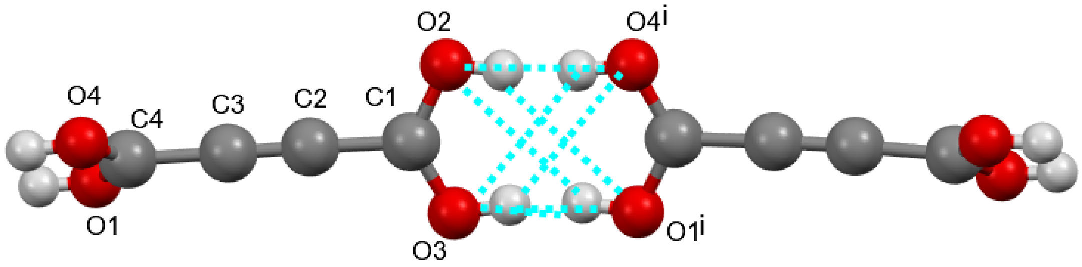

Figure S10: Presentation of molecules of acetylenedicarboxylic acid in anhydrous structure (ADCA) [32]. The lengths of bonds between carbon atoms in ADCA [(C2≡C3 1.188, C1−C2 1.454 and C3−C4 1.455 Å) are similar to that of ADAD. On the other hand, the bond lengths C1≈O2 1.262, C1≈O3 1.245, C4≈O1 1.259 and C4≈O4 1.246 Å and H atoms positions with 50% occupancy reflect that carboxyl groups in ADCA are disordered which is not the case in ADAD. The conformation of acid molecules in anhydrous structure also differs from that of hydrated. In ADAD molecules are centrosymmetric and nearly planar while in ADCA molecules are asymmetric and twisted with torsion angle O2-C1-C4-O4 of ≈ 60°. Hydrogen bonds between acid and water molecules in ADAD are significantly stronger then hydrogen bonds between acid molecules in ADCA (O3…O^{i} 2.656 and O2…$O4^{i}$ 2.694 Å, i: -1/2+x,1.5-y,1/2+z). The structure of ADCA was determined also at high pressures from 0.18 up to 5.2 GPa) [32]. At high pressure the structure of ADCA remains to be disordered, with twisted molecules and hydrogen bonds of moderate strength.
